# A Single Amino Acid Change in Nramp6 from Sedum Alfredii Hance Affects Cadmium Accumulation

**DOI:** 10.3390/ijms21093169

**Published:** 2020-04-30

**Authors:** Zhuchou Lu, Shuangshuang Chen, Xiaojiao Han, Jin Zhang, Guirong Qiao, Yugen Jiang, Renying Zhuo, Wenmin Qiu

**Affiliations:** 1 State Key Laboratory of Tree Genetics and Breeding, Chinese Academy of Forestry, Xiangshan Road, Beijing 100091, China; luzc9581@163.com (Z.L.); chenshuang@jaas.ac.cn (S.C.); hanxiaojiao1004@163.com (X.H.); gr_q1982@163.com (G.Q.); 2 The Research Institute of Subtropical Forestry, Chinese Academy of Forestry, Fuyang, Hangzhou 311400, China; 3 Institute of Leisure Agriculture, Jiangsu Academy of Agriculture Sciences, Nanjing 210014, China; 4 Biosciences Division, Oak Ridge National Laboratory, Oak Ridge, TN 37831, USA; zhangj1@ornl.gov; 5 Agricultural Technology Extension Center of Fuyang District, Hangzhou 311400, China; fuyangjyg@163.com

**Keywords:** Nramp6, Cadmium accumulation, site-directed mutagenesis, *Sedum alfredii* Hance

## Abstract

*SaNramp6* in *Sedum alfredii* encodes a membrane-localized metal transporter. We isolated the *SaNramp6h* allele from the hyperaccumulating ecotype (HE) of *S. alfredii*. When this allele was expressed in transgenic yeast and *Arabidopsis thaliana*, it enhanced their cadmium (Cd) sensitivity by increased Cd transport and accumulation. We isolated another allele, *SaNramp6n*, from a nonhyperaccumulating ecotype (NHE) of *S. alfredii*. Amino acid sequence comparisons revealed three amino acid differences between SaNramp6h and SaNramp6n. We investigated the Cd transport activity of the Nramp6 allele, and determined which residues are essential for the transport activity. We conducted structure-function analyses of SaNramp6 based on site-directed mutagenesis and functional assays of the mutants in yeast and *Arabidopsis*. The three residues that differed between SaNramp6h and SaNramp6n were mutated. Only the L157P mutation of SaNramp6h impaired Cd transport. The other mutations, S218N and T504A, did not affect the transport activity of SaNramp6h, indicating that these residues are not essential for metal selectivity. Transgenic plants overexpressing *SaNramp6h*^L157P^ showed altered metal accumulation in shoots and roots. Our results suggest that the conserved site L157 is essential for the high metal transport activity of SaNramp6h. This information may be useful for limiting or increasing Cd transport by other plant natural resistance associated macrophage protein (NRAMP) proteins.

## 1. Introduction

Cadmium (Cd) is a heavy metal with strong biological toxicity that is widely distributed in soil, water and the atmosphere. It causes great harm to animals and plants and can threaten human health [1]. Contamination of soil and water with Cd represents a major environmental problem [2]. Hyperaccumulator plants can grow in heavy metal-contaminated areas and accumulate high concentrations of heavy metals in their aboveground parts without toxicity symptoms [3]. Hyperaccumulator plants have potential applications in phytoremediation. Compared with nonaccumulator plants, hyperaccumulators show enhancement of one or more physiological mechanisms; for example, heavy metal uptake, root-to-shoot translocation, detoxification, and/or sequestration [4].

Plants respond to heavy metal stress by altering diverse genetic pathways. Such pathways include the synthesis of signaling molecules and stress-related proteins, such as antioxidant enzymes, metal chelators, osmotic regulators, and heavy metal transporters. These proteins function in the absorption, transport, intracellular transport, and detoxification of heavy metals, which are necessary for plants to maintain intracellular metal homeostasis. It is of great theoretical significance and practical value to study the physiological response of plants to Cd stress and the molecular mechanism of related genes and stress signal transduction pathways.

Heavy metal transporters play important roles in metal ion homeostasis, and include the cation diffusion facilitator (CDF) family [5], the heavy metal ATPase (HMA) family [6], the yellow-stripe 1-like (YSL) family [7,8], the ATP-binding cassette (ABC) family [9], the multidrug and toxic compound extrusion (MATE) family [10], the cation/H+ exchanger (CAX) family [11], zinc (Zn)-regulated transporters, the iron (Fe)-regulated transporter-like protein (ZIP) family [12], and the natural resistance associated macrophage protein (NRAMP) family [13]. The NRAMP family of proteins is evolutionarily conserved and functions as a symporter to transport diverse metals including iron (Fe), Zn, manganese (Mn), and Cd. Most of our knowledge about the mechanisms of these transporters is derived from studies on *Arabidopsis thaliana* and rice (*Oryza sativa*), whose NRAMPs play critical roles in Mn homeostasis and Cd toxicity [13,14,15,16,17,18]. Six NRAMP gene family members have been identified in *Arabidopsis*. Their encoded proteins include AtNRAMP1, which transports Mn, Fe, and Cd [15]; AtNRAMP3 and AtNRAMP4, which transport Mn and Fe [16]; and AtNRAMP6, which transports Cd [17]. In rice, OsNRAMP5 is a constitutively expressed protein essential for Fe, Mn, and Cd uptake [13], and knockout of *OsNramp5* was shown to reduce Cd accumulation in rice [18].

Previously, Zhang et al. (2015) identified five *NRAMP* genes from the transcriptome of the hyperaccumulating ecotype (HE) *Sedum alfredii* [19]. Among them, *Nramp6* encodes cell membrane-localized protein that is highly expressed in the shoot, where it plays a role in transporting Cd and Mn [19,20]. Transgenic *A. thaliana* expressing *SaNramp6h* exhibited high Cd accumulation levels. However, the structure-function relationship of SaNramp6h remains elusive to date. A previous study identified two *S. alfredii* genotypes, one of which was growing in the Southeast of China in an abandoned mine region with a high concentration of heavy metals in the soil [21]. *S. alfredii* (Crasulaceae) is the only known Cd-hyperaccumulating species that is not in the Brassica family [22]. The Cd concentrations in the leaves and stems of the hyperaccumulating ecotype (HE) reached 9000 and 6500 mg kg^−1^ (DW), respectively, when plants were supplied with 400 μmol Cd L^−1^ [23]; the nonhyperaccumulating ecotype (NHE) is not tolerant to heavy metals, and its leaf Cd concentrations reached only 533 mg/kg [24]. The reason for this difference is unknown.

In this study, we identified and analyzed *SaNramp6h* from the HE of *S. alfredii* and its homolog *SaNramp6n* from the NHE of *S. alfredii*. A sequence comparison analysis revealed three amino acid differences between *SaNramp6h* and *SaNramp6n*—Leu157Pro, Ser218Asn and Thr504Ala. We speculated that these differences in this homologous gene may be some of the reasons for the difference in Cd tolerance between the two genotypes. Each of the three amino acid residues that differ between *SaNramp6h* and *SaNramp6n* was altered by PCR site-directed mutation, and the effects of these mutations on Cd uptake and accumulation were test by expression in yeast and *A. thaliana*.

## 2. Results

### 2.1. Sequence Alignment and Expression Analyses of SaNramp6h and SaNramp6n

**Figure 1:**
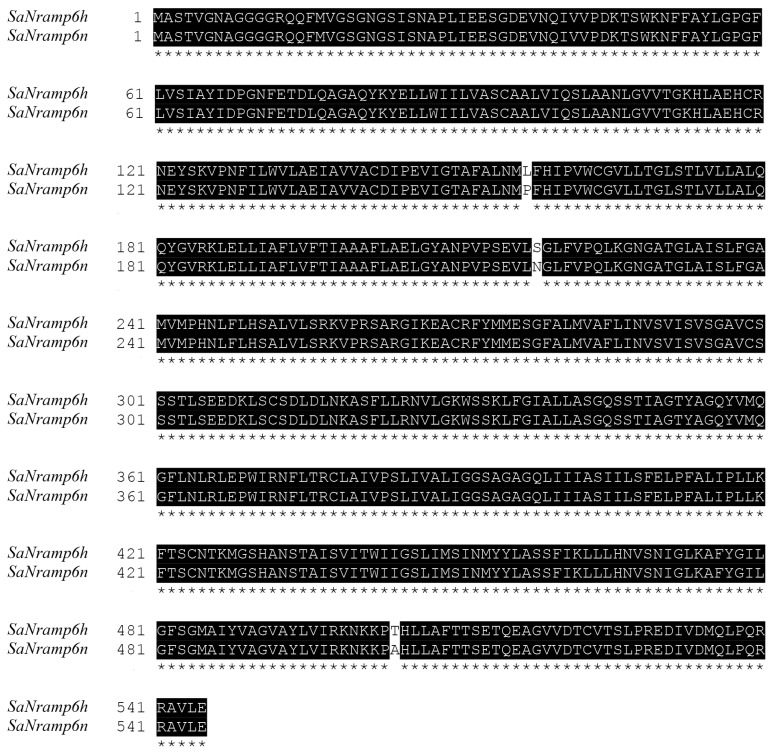
Amino acid sequence alignment of SaNramp6h and SaNramp6n. Sequence alignment was performed by ClustalX. Identical residues are in black and indicated symbol * at the bottom.

The coding regions of SaNramp6 from the two distinct ecotypes studied previously were sequenced and compared. This comparison showed that five nucleotides differ between *SaNramp6h* and *SaNramp6n* (Figure 1), corresponding to three amino acid differences between SaNramp6h and SaNramp6n at position 157 (Leu to Pro), 218 (Ser to Asn), and 504 (Thr to Ala). The difference at position 119 and 176 resulted from synonymous mutation.

**Figure 2:**
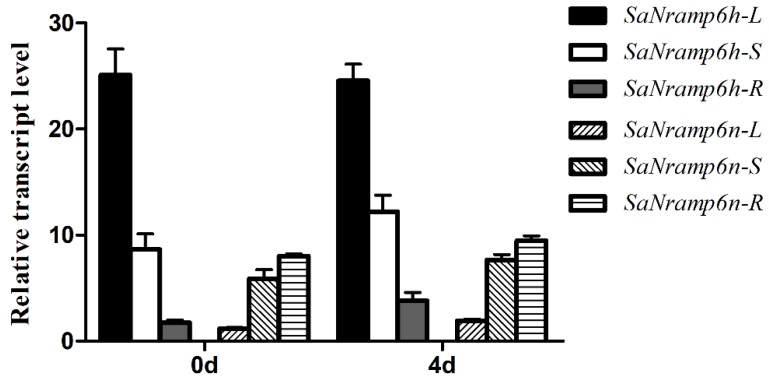
Expression patterns of *SaNramp6h* and *SaNramp6n* before and after treatment with cadmium (Cd). L, leaf; S, stem; R, root. Data are means ± SD (*n* = 9 replicates).

The transcript levels of the *SaNramp6* genes were compared between the two ecotypes using qRT-PCR analyses. There were no obvious differences in the transcript levels of *SaNramp6* in response to Cd treatment in either ecotype. The transcript levels were higher in shoots and lower in the roots in HE plants compared with the NHE plants. In HE plants, *SaNramp6* was primarily expressed in the leaves (Figure 2).

### 2.2. Functional Characterization of Nramp6 in ycf1 Yeast Mutant

**Figure 3:**
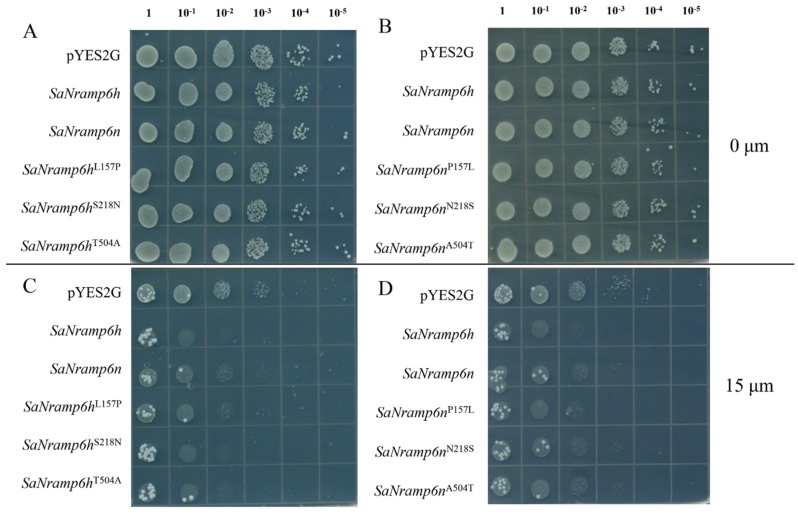
Metal toxicity growth assays of mutated NRAMP6 variants in *Saccharomyces cerevisiae* mutant ycf1. Yeast strain was transformed with EV (pYES2G empty vector) and other different mutations, and then grown on plates containing synthetic galactose-uracil (SG-U) without (**A**,**B**) or with 15 μM CdCl_2_ (**C**,**D**) for 3 days.

The sequence comparison showed that the vast majority of residues are conserved between *SaNramp6h* and *SaNramp6n*, suggesting that they are derived from a common ancestor, and raising the possibility that the residues differing between the two alleles represent candidate sites of functional divergence. The three *SaNramp6h*-specific residues (Nr1, Nr2, Nr3) were each substituted with the corresponding residues in *SaNramp6n* (Nr4, Nr5, Nr6) by PCR site-directed mutagenesis. The Cd transport activity of the resulting mutants was tested in a yeast mutant. YCF1(Yeast cadmium factor 1) is a member of the ABC transporter (ATP-binding cassette transporter) family, and is localized in the tonoplast. It is responsible for the transport of Cd ions to vacuoles and their detoxification by chelation with glutathione [25,26]. Therefore, the *ycf1* yeast mutant is sensitive to Cd at a certain concentration, making it a useful tool for identifying residues involved in Cd transport by SaNramp6. Heterologous expression of either native or mutant *SaNramp6* in yeast showed that both *SaNramp6h* and *SaNramp6n* could enhance the sensitivity of transgenic yeast to Cd, compared with that of the strain expressing the empty vector. However, ycf1 transformed with *SaNramp6n* exhibited a smaller degree of reduced growth (Figure 3). The S258N and T504A mutations did not affect the yeast’s sensitivity to Cd, but the L157P mutation in *SaNramp6h* (Nr1) led to a significantly decreased sensitivity to Cd. Thus, when the first amino acid residue of *SaNramp6n* was replaced with that of *SaNramp6h*, its phenotype became consistent with that of *SaNramp6h*, whereas there was no phenotypic difference resulting from the mutations at the other two residues. These results demonstrate the importance of Leu at positon 157 in the Cd transport activity of the protein.

**Figure 4:**
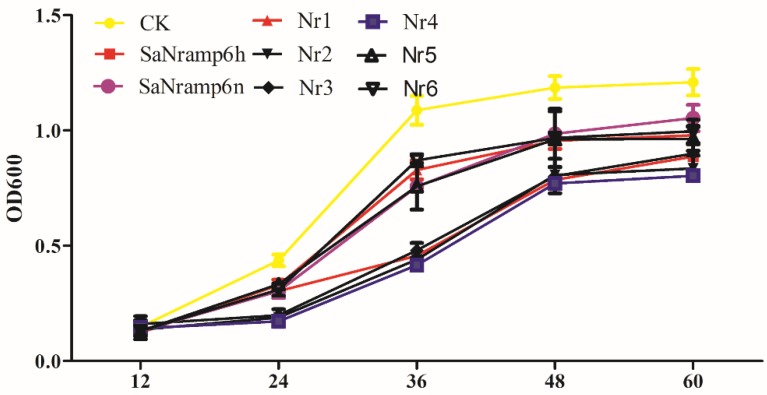
Growth curve of transgenic yeast under cadmium stress. Time-dependent growth of yeast strains in SG-U liquid medium supplemented with 5 μM CdCl_2_.

To confirm the involvement of residue 157 in Cd transport, we tested the ability of yeast strains expressing the mutant proteins to grow in the presence of 15 μM CdCl_2._ The strain expressing SaNramp6 showed lower growth rates than that of the negative control, consistent with the results of the spotting assay. Yeast cultures expressing mutated SaNramp6h^L157P^ exhibited similar or enhanced growth rates relative to that of its native counterpart. In contrast, the P to L mutation at position 157 in SaNramp6n showed significantly impaired growth (Figure 4). To further evaluate the effect of the mutations, we selected a period of logarithmic growth to measure the metal content in culture cells where results were consistent with the conclusions above (Supplemental Figure S1).

### 2.3. Phenotypic Analysis of Different Transgenic A. Thaliana Lines

**Figure 5:**
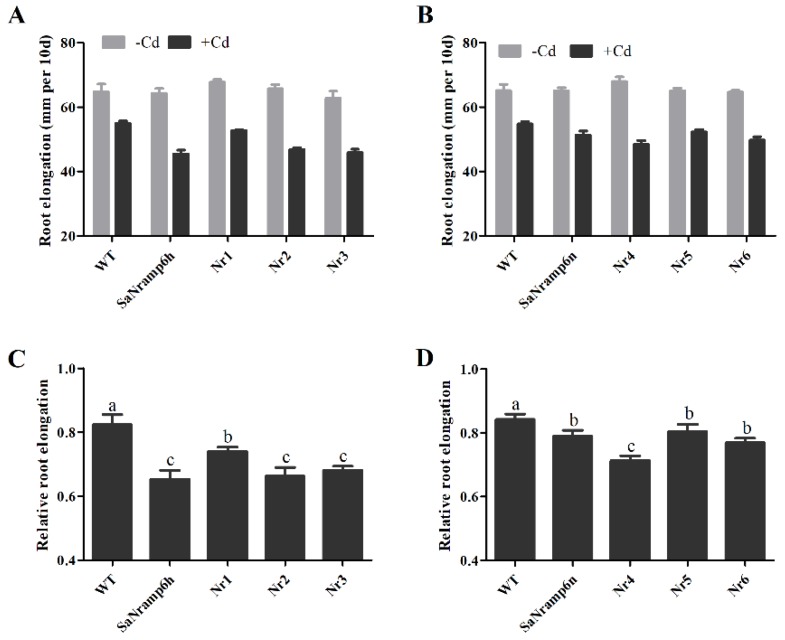
Relative primary root length of transgenic *Arabidopsis* lines expressing mutations of *SaNramp6h*. Growth of 14-day-old *Arabidopsis* Col-0 (wild type) and *Arabidopsis* lines overexpressing SaNramp6h (from the hyperaccumulating ecotype (HE)), SaNramp6n (from the nonhyperaccumulating ecotype (NHE)), or each of six mutations. Seedlings were germinated on ½MS medium and transferred to fresh medium supplemented with 50 μM CdCl_2_ for 5 d. (**A**,**B**) Primary root length. (**C**,**D**) Relative root growth (root growth of seedlings treated with 50 μM CdCl_2_ compared with root growth of untreated seedlings). The level of significance difference is indicated by the letter a, b, c. The bars with the same letters are not significantly different at relative root length at *P* < 0.05 according to Tukey’s test, respectively.

To explore the biological relevance of the SaNramp6 mutations in plants, *A. thaliana* seedlings of wild type (WT) and lines overexpressing *SaNramp6h*, *SaNramp6n*, and the mutations at Nr1, Nr2, Nr3, Nr4, Nr5, and Nr6 were germinated on ½-strength Murashig and Skoog (½MS) medium and then transferred to fresh medium with or without 50 μM CdCl_2_. Relative primary root (PR) growth of the seedlings was compared between the control (no Cd) and the Cd-containing medium. The results showed that the relative PR length of transgenic *Arabidopsis* expressing native *SaNramp6* and other variants was significantly inhibited. The reduction in root growth of the lines expressing Nr2 and Nr3 was similar to that of the line expressing *SaNramp6h*. The root length of the line expressing Nr1 was greater than that of the line expressing *SaNramp6h*. The lines expressing *Nr5* and *Nr6* had the same inhibited root growth response as *SaNramp6n*, whereas the line expressing *Nr4* showed very strong inhibition of root growth (Figure 5). Fresh weight and chlorophyll content behaved in a similar manner (Supplemental Figure S2). These results were consistent with those of the plate-based toxicity assay in yeast. We attributed impaired root growth to toxic intracellular accumulation of Cd.

**Figure 6:**
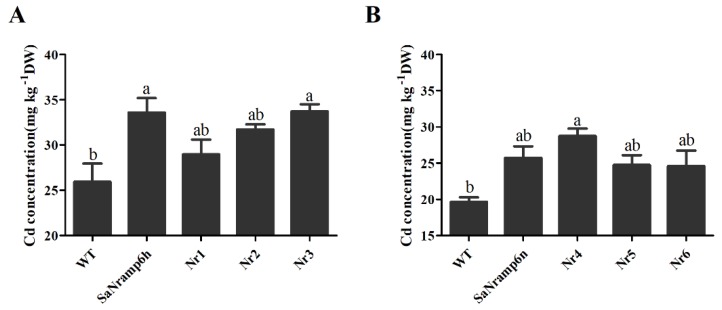
Cadmium (Cd) accumulation in transgenic *Arabidopsis* lines. Four-week-old hydroponically grown Col-0 (wild type, WT) and lines overexpressing SaNramp6h (from HE), SaNramp6n (from NHE) and six other mutations were transferred to fresh medium containing 30 µM CdCl_2_ and grown for 7 d before measuring Cd contents. (**A**) The Cd concentration in WT, SaNramp6h, Nr1, Nr2 and Nr3 mutations. (**B**) The Cd concentration in WT, SaNramp6n, Nr4, Nr5 and Nr6 mutations.

To further investigate the underlying cause of the pronounced difference in Cd transport between native SaNramp6 and the L157P mutants, we determined metal concentrations in the *Arabidopsis* lines expressing SaNramp6 and the various mutations in the presence of 30 μM CdCl_2_. The L157P mutation in Nr1 impaired Cd transport by SaNramp6h, while the S218N mutation in Nr2 and the T504A mutation in Nr3 did not affect the Cd transport function of the protein. However, the concentrations of other mineral nutrients such as Fe and Mn did not differ between the lines (Supplemental Table S1). These results provided further evidence that Leu 157 is involved in Cd transport. The Cd concentration was higher in the line overexpressing Nr4 than in the lines overexpressing *SaNramp6n*, Nr5, and Nr6 (Figure 6), further demonstrating the importance of Leu 157 in Cd accumulation.

## 3. Discussion

Genes encoding NRAMPs have been identified in a wide variety of organisms, from bacteria to humans [27]. They play important roles in metal ion homeostasis and are usually highly expressed in hyperaccumulating plants [28]. Previous studies have detailed the selective and molecular transport mechanisms of plant NRAMP proteins for heavy metal ions. Some reports have described enhanced heavy metal transport and accumulation by NRAMP proteins in hyperaccumulator plants. However, the differences in NRAMP proteins between hyperaccumulator plants and nonhyperaccumulator plants remain poorly understood. SaNramp6 belongs to the NRAMP family, which is crucial for metal homeostasis, especially for Fe, Mn, and Cd. Sequence and phylogenetic analyses showed that SaNramp6 contains 11 transmembrane domains and most closely resembles AtNramp6, which is a plasma membrane-localized Cd transporter [17]. In the present study, SaNramp6h and SaNramp6n were found to exhibit different transport activity. *SaNramp6h* from HE *S. alfredii*, when expressed in yeast and *A. thaliana*, could transport Cd more effectively than could *SaNramp6n* from NHE *S. alfredii*.

So far, in addition to *S. alfredii*, four other species have been identified as Cd hyperaccumulators*—Arabidopsis halleri* [29], *Thlaspi praecox* [30], *Thlaspi caerulescens* [31], and *Solanum nigrum L.* [32]. Compared with nonhyperaccumulating ecotypes, Cd hyperaccumulator plants show enhanced Cd uptake from soil into root cells, highly efficient Cd loading into the xylem, enhanced Cd transport from the roots to shoots, and stronger detoxification of Cd in the aboveground area [33].

Generally, the absorption of Cd by higher plants is mainly through the competitive entry via transporters of divalent cations such as Ca, Zn, Fe, and Mn. In the hyperaccumulator plants *A. halleri* and *T. praecox*, an increase in the exogenous Zn concentration can reduce Cd uptake, indicating that Cd transport depends on a Zn transporter that has a higher affinity for Zn than for Cd [34]. However, in *N. caerulescens*, the hyperaccumulating ecotype (Ganges) may have a high affinity transport system for Cd that is independent of Zn transport, because its Cd uptake is not affected by exogenous Zn [35]. It has been demonstrated that hyperaccumulators evolve from nonhyperaccumulators under long-term high-level heavy metal conditions [36,37]. During this evolutionary process, transporters may gradually change in their ability to transport metals such as Cd, Zn, and Pb. Heterologous expression of SaHMA3 in *S. cerevisiae* and tobacco showed that SaHMA3h from HE *S. alfredii* can only transport Cd, while SaHMA3n from NHE *S. alfredii* has both Cd and Zn transport activity [38]. In *N. caerulescens*, NcHMA3 is a specific Cd transporter, but it shows different affinity for Cd among different genotypes [33]. It is interesting that these homologous genes from different plants have evolved a similar ability to enhance transport activity.

Transporter proteins encoded by different alleles of the gene in different plant genotypes can exhibit different substrate affinity. This provides a useful tool for identifying critical residues for metal transport activity and selectivity. Most residues are conserved so that protein maintains its activity, but some residues responsible for ion selectivity can change without altering protein function. In *Arabidopsis* AtNRAMP3, the M248A mutant retains Mn transport activity but showed severely reduced Cd transport activity, which decreases Cd sensitivity [39]. Similarly, some mutations in AtNRAMP4 selectively modify Cd^2+^ and Zn^2+^ accumulation without affecting Fe transport [40]. In our study, both *SaNramp6h* and *SaNramp6n* showed some similarities in function but differed in their Cd selectivity and expression patterns. In *SaNramp6h*, the Cd transport activity was significantly decreased by the L157P mutation, but increased by the P157L mutation. However, the S218N and T504A mutations did not affect its transport activity. This indicates that during long-term evolution, residue L157 in *SaNramp6h* has become responsible for its specific affinity to Cd. Proline is most often thought of as the most rigid residue because of its rigid pyrrolidine ring, and introduction of proline can produce a protein conformational change. Proline substitution at position 157 would enhance the stability of SaNramp6 protein. However, the transport activity of the L157P mutant is decreased relative to that of native SaNramp6h. We infer that L157P might have influenced Cd binding sites of SaNramp6. The discordance between transport activity and stability of the protein needs to be further studied.

The expression pattern of *SaNramp6* differed significantly between the HE and NHE plants. The transcript levels of *SaNramp6* were significantly higher in HE plants than in NHE plants. The *SaNramp6* was constitutively expressed in the shoots. In NHE plants, the transcript levels of *SaNramp6* were slightly higher in the roots than in the shoots. Similar results have been reported for the hyperaccumulating ecotype (Ganges) of *N. caerulescens*, based on a comparison of the transcriptomes between two ecotypes. Further research showed that NcNramp1 participates in the transport of Cd into the stele and from the root to shoot. Increased expression of several genes encoding metal transporters was identified as the main reason for Cd hyperaccumulation in *N. caerulescens* [41]. In the case of the expression of Nramp6 under the 35S promoter, such as in the present study, all the plant organs accumulated Cd and significant sensitization might be expected because of incorrect tissue specificity. However, in the HE plants, Nramp6 seems to function as an enhancer for improved shoot internal sequestration in combination with the buffering of physiologically available Cd in the cytosol or efflux into the vacuole of the shoot cell. Thus, our data highlight two aspects of the roles of *SaNramp6* in Cd sequestration—elevated transcript levels in the shoots may be associated with high Cd accumulation in shoots, and lower expression levels in the roots may explain why less Cd is retained in the roots.

## 4. Materials and Methods

### 4.1. Yeast Strain and Culture Conditions

The Cd-sensitive *S. cerevisiae* strain BY4742Δ*ycf1* (MATα; his3Δ1; leu2Δ0; met15Δ0; ura3Δ0; YDR135c::kanMX4) was used in this study. Yeast cells were transformed by electroporation [42]. Positive transformants were selected on synthetic defined medium lacking uracil (SD-U), containing 2% (*w/v*) glucose.

### 4.2. Gene Cloning and Expression Analysis

Total RNA was extracted from root, stem and leaf tissues of *S. alfredii* seedlings using a Plant Tissue Total RNA Purification Kit (NORGEN, Thorold, Canada) according to the manufacturer’s instructions. The reverse transcription reactions were performed using a Superscript RT III first-strand cDNA synthesis kit followed by treatment with RNase H (Invitrogen, Carlsbad, CA, USA). The CDS fragments of *SaNramp6n* were amplified by high fidelity KOD-Plus DNA Polymerase (Toyobo, Osaka, Japan) using the primer, which were originally used to amplify the *SaNramp6h* allele [20]. The amino acid sequences of *SaNramp6h* and *SaNramp6n* were compared using ClustalX.

To evaluate the effect of Cd on the expression of *SaNramp6*, *S. alfredii* HE and NHE plants were treated with 100 μM CdCl_2_ for 4 days. The plants were divided into leaves, stem, and roots, which were immediately frozen in liquid nitrogen before extracting RNA. The *SaNramp6* expression levels were quantified using PrimeScript^TM^ RT SYBR Green Master Mix (Takara, Dalian, China). The transcript levels were calculated relative to that of *SaUBC9* as the reference gene [20].

### 4.3. Site-Directed Mutagenesis and Construction of Expression Vector

The cDNAs of *SaNramp6h* and *SaNramp6n* were reciprocally mutagenized by a PCR-mediated site-directed mutation technique [43]. First, single mutants were introduced into the primers B1 and B2. To facilitate the amplification of the target fragment, we added the entry vector connector sequence before the full-length primer. The upper half of the target fragment was amplified using the full-length upstream primer A1 and the downstream primer B1, and then the lower half of the target fragment was amplified by the full-length downstream primer A2 and the upstream primer B2. Finally, the full-length primers A1 and A2 and the products recovered from the upper and lower segments were used as templates to amplify the complete target fragments. The purified full-length PCR fragment was inserted into the Gateway entry vector pENTR/D-Topo (Invitrogen, Carlsbad, CA, USA), then LR-recombined into the yeast expression vector pYES2.0 or the plant expression vector pH2GW7.0 [44]. Recombinant plasmids carrying native and mutated *SaNramp6h* and *SaNramp6n* were introduced into the yeast strain. The empty plasmid pYES2G was transformed into yeast cells as the negative control. Diluted cultures of individual transformants were spotted onto synthetic galactose-uracil (SG-U) agar plates containing 0 or 15 μM CdCl_2_. The plates were incubated at 30 °C for 3 days before growth phenotypes were evaluated. The yeast strains were also grown overnight in 10 mL minimal medium and then transferred to 100 mL SG-U containing 5 μM CdCl_2_, and the relative growth of the transformants was determined by measuring the OD_600_ every 6 h. The yeast cells were harvested for 36 h and washed with PBS before being dried and digested with a concentrated acid mixture of HNO3, HClO4, and H2SO4 (volume ratio = 4:1:0.5) at 250 °C for 8 h. The determination of Cd content via Inductively Coupled Plasma-Mass Spectrometry (ICP-MS) (NexION 300; PerkinElmer, Shelton, CT, USA).

### 4.4. Plant Materials and Growth Conditions

The HE *S. alfredii* plants originated from an old Pb/Zn mining region near the city of Quzhou in Zhejiang province, P. R. China. The NHE plants were obtained from a tea plantation in Hangzhou, Zhejiang province, P. R. China. The plant materials were cutting clones of the original plants, and were grown in half-strength Hoagland-Arnon solution, at 25 °C under a 16 h light/8 h dark photoperiod in an artificial climate incubator. The Columbia ecotype of *A. thaliana* from our laboratory was used for Agrobacterium-mediated transformation with pH2GW7.0-*SaNramp6* or *SaNramp6* point mutants.

### 4.5. Acquisition of Transgenic Arabidopsis and Cadmium Stress Treatment

The recombinant plasmid pH2GW7.0-*SaNramp6* was transformed into *Agrobacterium tumefaciens* EHA105, and the positive strain was then used to transfect *Arabidopsis* via the floral dip method [45,46]. Three hygromycin-resistant *Arabidopsis* lines were obtained after transformation with the *SaNramp6* construct. Transgenic lines that were PCR positive (Supplemental Figure S3) with similar expression levels of SaNramp6 (Supplemental Figures S4 and S5) were used for subsequent experiments. Seeds from one homozygous line T3 and the WT were placed on MS agar plates containing 50 μM CdCl_2_ using a sterilized toothpick. After vertical cultivation for 14 days in a greenhouse at 22 °C, roots were scanned using an Epson Perfection V700 Photo scanner (Seiko Epson Corp., Nagano, Japan). The length of roots was determined by WinRHIZO PRO2012a (Regent Instruments Inc., Quebec, Canada), for comparison of root growth among the WT and transgenic lines (Supplemental Figure S6).

The hydroponic system was based on the modified system of Chen et al. (2017) [20]. The seeds of WT and different transgenic *A. thaliana* lines were cultured in ½ MS solid medium for 1 week, and then grown in 1/4 Hoagland’s nutrient solution for 14 days under hydroponic conditions. Subsequently, the seedlings were treated with 30 mM CdCl_2_ for 7 days. All the seedlings were collected and fresh weights were determined. Chlorophyll was extracted by 90% acetone.

### 4.6. Element Content Analysis

For analyses of metal content in plants, roots and shoots were harvested separately. The roots were chelated with 1 mM EDTA for 30 min and then rinsed with distilled water three times. The dried samples were ground and subsequently digested in nitric acid. The Fe, Mn, and Cd concentration was determined by Inductively Coupled Plasma-Mass Spectrometry (ICP-MS) (NexION 300; PerkinElmer, Shelton, CT, USA).

## 5. Conclusions

*SaNramp6* from *S. alfredii* is a transport protein localized to the plasma membrane. Compared with *SaNramp6n*, *SaNramp6h* shows higher affinity for Cd. Amino acid residue L157 was identified as being critical for the Cd transport activity of *SaNramp6h*. The differences in the ionic selectivity and expression of the Nramp6 transporters between hyperaccumulating and nonhyperaccumulating ecotypes provide important clues about the mechanisms of Cd hyperaccumulation and tolerance in the hyperaccumulator *S. alfredii*.

## Supplementary Materials

The following are available online at https://www.mdpi.com/1422-0067/21/9/3169/s1, Table S1: Elemental concentrations in shoots and roots of wild-type and transgenic Arabidopsis lines expressing mutations of *SaNramp6*, Figure S1: Cd content of ∆ycf1 yeast cells expressing mutations of *SaNramp6*, Figure S2: Biomass analyses and chlorophyll content of wild-type and transgenic Arabidopsis lines expressing mutations of *SaNramp6*, Figure S3: PCR identification of transgenic *Arabidopsis thaliana* lines, Figure S4: RT-PCR analysis of the *SaNramp6* transcript in the wildtype (WT) and six site mutant mutations plants, Figure S5: Relative expression of *SaNramp6* in transgenic Arabidopsis lines expressing mutations of *SaNramp6*, Figure S6: Phenotypes of wild-type (WT) and overexpressing *SaNramp6h*, *SaNramp6n* and other six mutations of *Arabidopsis thaliana* under Cd stress.

## Abbreviations

Cd	Cadmium
Nr1	*SaNramp6h* ^L157P^
Nr2	*SaNramp6h* ^S218N^
Nr3	*SaNramp6h* ^T504A^
Nr4	*SaNramp6n* ^P157L^
Nr5	*SaNramp6n* ^N218S^
Nr6	*SaNramp6n* ^A504T^
L	Leucine
S	Serine
T	Threonine
P	Proline
N	Asparagine
A	Alanine
