# Supplementary material for: A Single Amino Acid Change in Nramp6 from Sedum Alfredii Hance Affects Cadmium Accumulation
Source: Int J Mol Sci. 2020 Apr 30;21(9):3169. doi: 10.3390/ijms21093169 (PMC7246828; doi:10.3390/ijms21093169)
Supplement: Supplementary file 1 [file ijms-21-03169-s001.pdf]

International Journal of
Molecular Sciences

MDPI

Supplemental Table S1. Elemental concentrations in shoots and roots of wild-type and transgenic Arabidopsis lines expressing mutations of *SaNramp6*

Genotype	Fe (g/Kg DW)		Mn (mg/Kg DW)	
	Shoot	Root	Shoot	Root
WT	0.144±0.046	1.51±0.24	0.206±0.024	1.012±0.21
Nrh	0.15±0.053	1.6±0.34	0.192±0.009	1.025±0.19
Nr1	0.146±0.043	1.503±0.17	0.181±0.029	1.058±0.23
Nr2	0.138±0.038	1.478±0.58	0.197±0.015	1.001±0.2
Nr3	0.153±0.015	1.493±0.46	0.213±0.027	1.055±0.15
Nrn	0.131±0.03	1.503±0.41	0.217±0.034	1.487±0.37
Nr4	0.13±0.051	1.357±0.19	0.204±0.048	1.08±0.22
Nr5	0.141±0.04	1.41±0.24	0.233±0.042	1.017±0.23
Nr6	0.13±0.062	1.543±0.12	0.214±0.071	1.063±0.24

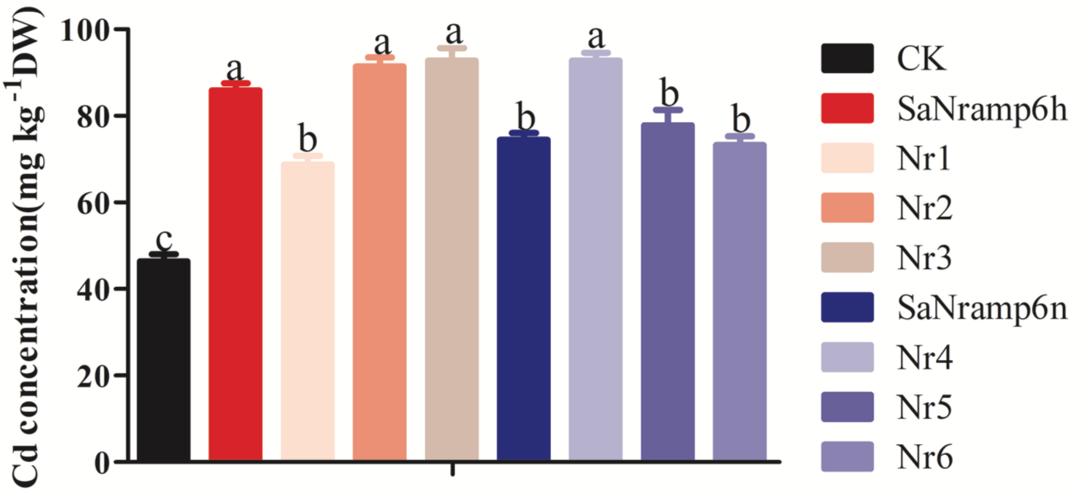

Supplemental Figure S1.

Cd content of Δ*ycf1* yeast cells expressing mutations of *SaNramp6* grown for 48 h in liquid SG-U supplemented with 5 µM CdCl2. Bars indicate means ± standard deviations (SDs) of at least three independent biological experiments.

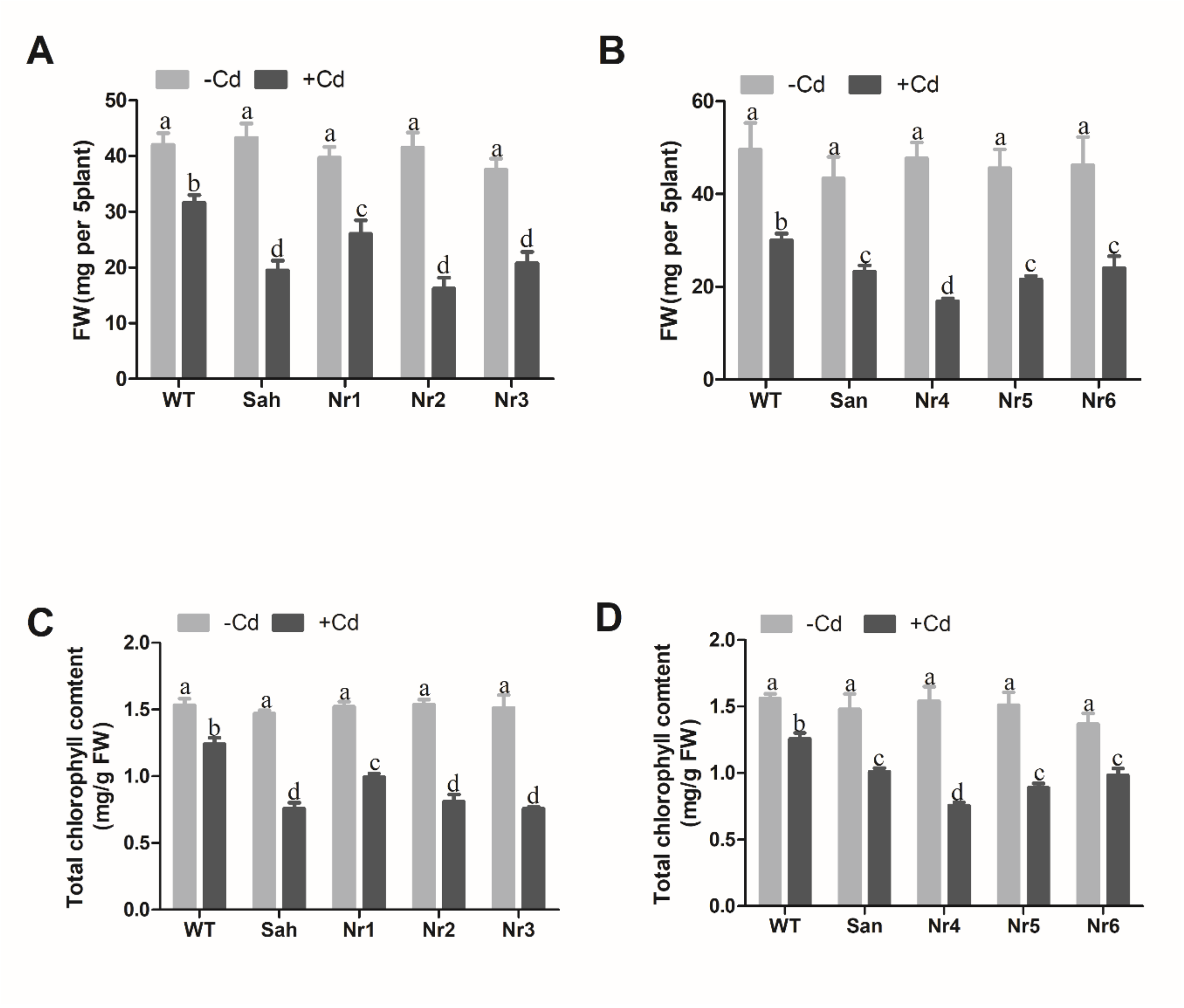

Supplemental Figure S2.

Biomass analyses and chlorophyll content of wild-type and transgenic Arabidopsis lines expressing mutations of *SaNramp6*. (A-B) Biomass assay. Biomass analyses of shoot. (C-D) Determination of total chlorophyll (chlorophyll a and chlorophyll b) content. Different small letters indicate significant differences ($p<0.05$) between treatments.

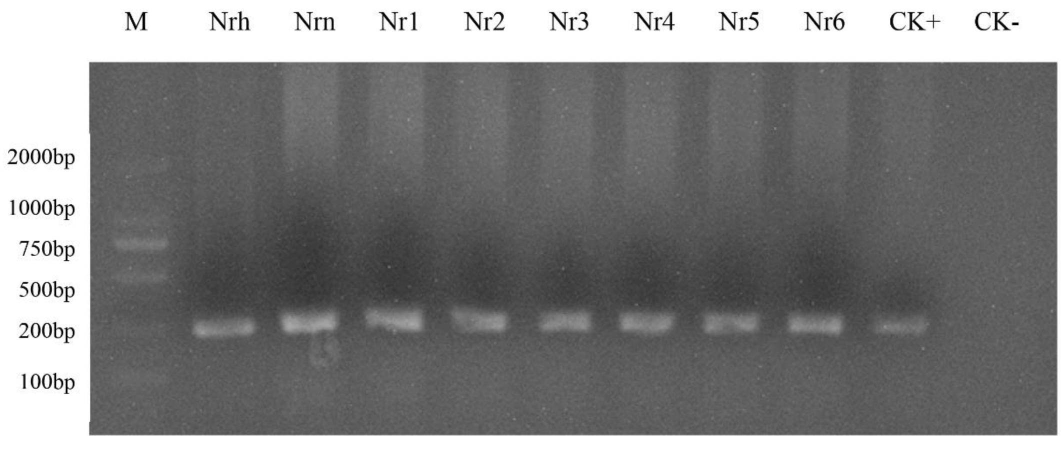

Supplemental Figure S3.

PCR identification of transgenic *Arabidopsis thaliana* lines. *SaNramp6h*, *SaNramp6n* (named Nrh and Nrn in figure). Positive plasmids and WT used as positive and negative control.

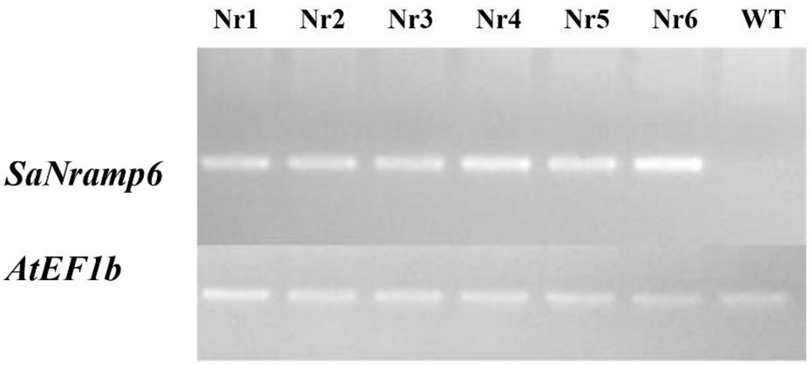

Supplemental Figure S4.

RT-PCR analysis of the *SaNramp6* transcript in the wildtype (WT) and six site mutant mutations plants. *AtEF1b* was used as the internal control.

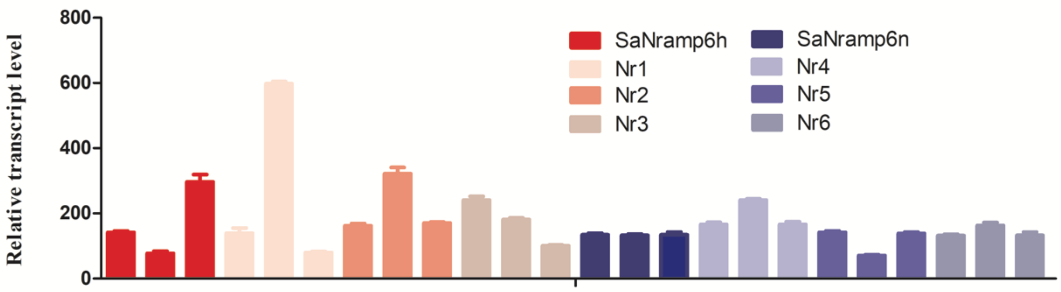

Supplemental Figure S5.

Relative expression of *SaNramp6* in transgenic Arabidopsis lines expressing mutations of *SaNramp6*. Expression levels were relative to Actin. Data are mean ± SD, n=3.

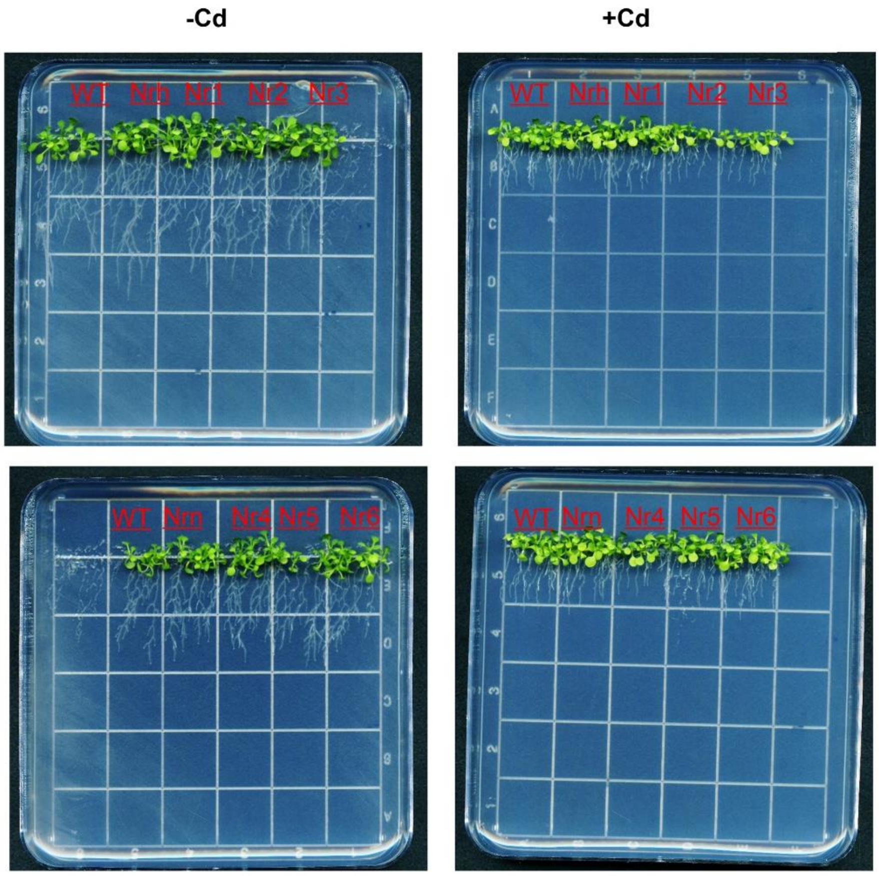

Supplemental Figure S6.

Phenotypes of wild-type (WT) and overexpressing *SaNramp6h*, *SaNramp6n* (named Nrh and Nrn in figure) and other six mutations of *Arabidopsis thaliana* under Cd stress. Five-day-old plants grown on half-strength Murashige and Skoog's (1/2 MS) medium were transferred to 1/2 MS medium without or with 50μM $CdCl_2$. Photographs were taken 12 days after the transfer.
